# Microbial community compositions in different functional zones of Carrousel oxidation ditch system for domestic wastewater treatment

**DOI:** 10.1186/s13568-017-0336-y

**Published:** 2017-02-15

**Authors:** Dong Xu, Sitong Liu, Qian Chen, Jinren Ni

**Affiliations:** 1 Department of Environmental Engineering, Peking University, Beijing, 100871 China; 2 The Key Laboratory of Water and Sediment Sciences, Ministry of Education, Beijing, 100871 China; 3 State Key Laboratory of Plateau Ecology and Agriculture, Qinghai University, Xining, 810016 Qinghai China

**Keywords:** Carrousel oxidation ditch, Biologically functional zone, Wastewater treatment, Microbial community structure, Illumina high-throughput sequencing

## Abstract

The microbial community diversity in anaerobic-, anoxic- and oxic-biological zones of a conventional Carrousel oxidation ditch system for domestic wastewater treatment was systematically investigated. The monitored results of the activated sludge sampled from six full-scale WWTPs indicated that *Proteobacteria*, *Chloroflexi*, *Bacteroidetes*, *Actinobacteria*, *Verrucomicrobia*, *Acidobacteria* and *Nitrospirae* were dominant phyla, and *Nitrospira* was the most abundant and ubiquitous genus across the three biological zones. The anaerobic-, anoxic- and oxic-zones shared approximately similar percentages across the 50 most abundant genera, and three genera (i.e. uncultured bacterium *PeM15*, *Methanosaeta* and *Bellilinea*) presented statistically significantly differential abundance in the anoxic-zone. Illumina high-throughput sequences related to ammonium oxidizer organisms and denitrifiers with top50 abundance in all samples were *Nitrospira*, uncultured *Nitrosomonadaceae*, *Dechloromonas*, *Thauera*, *Denitratisoma*, *Rhodocyclaceae* (*norank*) and *Comamonadaceae* (*norank*). Moreover, environmental variables such as water temperature, water volume, influent ammonium nitrogen, influent chemical oxygen demand (COD) and effluent COD exhibited significant correlation to the microbial community according to the Monte Carlo permutation test analysis (*p* < 0.05). The abundance of *Nitrospira*, uncultured *Nitrosomonadaceae* and *Denitratisoma* presented strong positive correlations with the influent/effluent concentration of COD and ammonium nitrogen, while *Dechloromonas*, *Thauera*, *Rhodocyclaceae* (*norank*) and *Comamonadaceae* (*norank*) showed positive correlations with water volume and temperature. The established relationship between microbial community and environmental variables in different biologically functional zones of the six representative WWTPs at different geographical locations made the present work of potential use for evaluation of practical wastewater treatment processes.

**Electronic supplementary material:**

The online version of this article (doi:10.1186/s13568-017-0336-y) contains supplementary material, which is available to authorized users.

## Introduction

Oxidation ditches are widely applied in wastewater treatment, especially in the small-scale and medium-scale wastewater treatment plants (WWTPs) in China (Zhang et al. 2016). As a modified activated sludge process, oxidation ditches have distinct advantages in simple construction, flexible operating mode, low sludge production, and special capability of nitrification and denitrification within the same tank. An oxidation ditch is often used to achieve simultaneous nitrification and denitrification because of the alternation of aerobic and anoxic zones within a channel, which can be formed by regulating the oxygen supply with surface aeration equipment or surface aeration combined with microporous aeration (Ammary and Radaideh 2005; Liu et al. 2010; Jin et al. 2015; Zhou et al. 2015).

The Carrousel oxidation ditch system, usually with an external pre-anaerobic zone and an internal pre-anoxic zone, is one of the most commonly used type of oxidation ditches (Peng et al. 2008; Jin et al. 2014). Carrousel oxidation ditch is designed to achieve an excellent condition for denitrifying and nitrifying bacteria in alternating anoxic–oxic zones in the looped channel system. In recent decades, operational conditions (e.g. temperature, hydraulic retention time, dissolved oxygen and operational mode), ditch geometry, aerator design and mode of aeration have been extensively investigated to optimize the treatment process and enhance nitrogen removal (Liu et al. 2010; Saida et al. 2010; Jin et al. 2015). Meanwhile, mathematical models have also been developed for simulating hydrodynamics, oxygen mass transfer, carbon oxidation, nitrification and denitrification processes, and optimizing the complicated alternating aerobic and anoxic conditions in oxidation ditches (Saida et al. 2010; Xie et al. 2011; Yang et al. 2013; Lei and Ni 2014).

As an important microbially mediated process for wastewater treatment and nitrogen removal, the efficiency and stability of Carrousel oxidation ditch system is entirely dependent upon the concerted and syntrophic activity of microorganisms belonging to different function performing nitrification, anammox and denitrification (Vanwonterghem et al. 2014; Rodríguez et al. 2015). Recently, several molecular technologies based on microbial 16S rRNA have been used to investigate the microbial community structures in oxidation ditch system (Zheng et al. 2015; Xia et al. 2016). For instance, Guo et al. (2013) studied the community structure of the nitrifying bacteria using fluorescence in situ hybridization (FISH). Zhou et al. (2015) identified the simultaneous nitrification and denitrification in an oxidation ditch by observing coexistence of nitrifying and denitrifying bacteria as well as some microaerophilic microorganisms using FISH and polymerase chain reaction–denaturing gradient gel electrophoresis methods. Jin et al. (2015) investigated the effect of different aeration modes on microbial communities of a Carrousel oxidation ditch by high-throughput 454 Pyrosequencing, of which activated sludge samples were sampled from anoxic and oxic zones and mixed with same proportion.

In this study, Illumina high-throughput sequencing was employed to reveal the microbial community diversity and structure of the activated sludge sampled from six full-scale WWTPs with Carrousel oxidation ditch systems. The core microbial populations and distribution of ammonium oxidizer organisms and denitrifiers in different functional zones in Carrousel oxidation ditch systems were studied. Differentially abundant features of the core microorganisms between the three biological functional zones and between the six different geographically located WWTPs were evaluated. More importantly, relationship between microbial community and environmental variables were established, which is of significance to diagnosis of practical wastewater treatment processes.

## Materials and methods

### Sample collection and determination

The investigated six full-scale Carrousel oxidation ditch systems (all equipped with an external pre-anaerobic zone and an internal pre-anoxic zone) respectively belong to six WWTPs at geographically different location in China, represented by XJYQ, ZZLQ, HBLY, BJYF, GDHZ and MYYX. The descriptions of these WWTPs were shown in Additional file 1: Table S1. The anaerobic-(A1), anoxic-(A2) and oxic-(O) activated sludge samples were collected respectively from the corresponding biologically functional zones in Carrousel oxidation ditch systems. Each activated sludge sample was fixed on site by mixing with 50% ethanol (v/v) on site, kept in an ice box for transport and stored at −20 °C in laboratory before DNA extraction. Concentrations of chemical oxygen demand (COD) and ammonium nitrogen in the influent/effluent of the WWTPs were measured according to standard analytical procedures (Clesceri et al. 1998). The level of pH and DO was determined on site by a pH sensor (pHS-25) and a DO sensor (WTW Oxi 340i), respectively.

### DNA extraction

Genomic DNA was extracted from each activated sludge sample using the PowerWater DNA Isolation Kit (MO BIO, CA, USA) according to manufacturer’s protocols. The extracted DNA samples were stored at −20 °C for subsequent assays. The intact DNA was confirmed on 1.5% agarose gel electrophoresis. The concentration and quality of the extracted DNA were assessed with a Nanodrop2000 microspectrophotometry (Thermo Scientific, DE, USA).

### Polymerase chain reaction (PCR) amplification and high-throughput sequencing

The hypervariable V3–V4 region of 16S rRNA genes were amplified from all DNA extracts with barcoded primers 340F (CCTACGGGNBGCASCAG) and 805R (GACTACNVGGGTATCTAATCC) under following conditions: initial denaturation at 95 °C for 3 min, followed by 30 cycles at 95 °C for 30 s, 50 °C for 30 s and 72 °C for 60 s and final extension at 72 °C for 7 min by the LabCycler PCR (Sensoquest, Germany). The 50 µL PCR mixture contained 5 µL of 10× buffer, 38.8 µL of 5 ddH_2_O, 1 µL of dNTP (10 mM), 2 µL of each primer, 0.2 µL of KAPA Taq polymerase (KAPA Biosystems, USA) and 1 µL of genomic DNA. After confirmed by 1.5% agarose gel electrophoresis, the PCR products were mixed to get equal concentration of DNA fragment for each sample and purified using MinElute PCR Purification Kit (QIAGEN, Germany), and then were sequenced using the Illumina Hiseq2500 PE250 platform.

### High-throughput sequencing data analysis

All the obtained paired-end reads of 16S rRNA gene PCR amplicons were quality filtered and denoised to remove low quality or ambiguous reads. Then the treated forward and reverse reads were merged with PANDAseq (Masella et al. 2012). The putative chimeric sequences were identified and excluded with USEARCH61 pipeline (Edgar et al. 2011) in QIIME (Caporaso et al. 2010). The remaining set of high quality sequences were clustered into operational taxonomic units (OTUs) at a 97% similarity threshold using UCLUST methods (Edgar 2010) embedded in QIIME. The taxonomic identities of the representative sequences from each OTU were classified via the Ribosomal Database Project (RDP) classifier with the SILVA databases (Wang et al. 2007). The Illumina sequencing raw data obtained from this study were deposited in the NCBI Sequence Read Archive with accession No. SRP093686 (PRJNA354474).

### Statistical analyses

Heatmap of the top 50 genera in each sample was conducted using R packages. The open source software Cytoscape v3.3.0 (Shannon et al. 2003) was employed for clustering network analysis to visualize the most abundant OTUs and to compare their abundance among the different samples. The linear discriminant analysis (LDA) effect size (LEfSe) pipeline (http://huttenhower.sph.harvard.edu/galaxy/) (Segata et al. 2011) was used to identify differentially abundant features among the different biological functional zones of Carrousel system and different sampling WWTPs. The differential features were identified on the OTU level (relative abundance >1%). The nonparametric factorial Kruskal–Wallis (KW) rank sum test was used to detect taxa with significant differential abundances. LDA was used to evaluate the effect size of each differentially abundant trait. The LEfSe analysis was performed under the alpha value for the Kruskal–Wallis test is <0.05, and the threshold on the logarithmic LDA score for discriminative features is >2.0 (Zhang et al. 2013). Principal component analysis (PCA) was conducted using Canoco 4.5 (Microcomputer Power, USA). Redundancy analysis (RDA), a form of constrained ordination, was employed to analyze the relationships between the abundance of core genera and environmental variables using R software with the vegan and ggplot2 packages.

## Results

### Biodiversity and microbial community profiles in Carrousel system

Using Illumina Sequencing of 16S rRNA Gene Amplicons, 8017-10681 OTUs obtained from the individual activated sludge sample. The OTUs, Good’s coverage, Chao1, ACE, Shannon and Simpson of each sample could be seen in Additional file 1: Table S2. Forty-eight bacterial and 7 archaeal phyla were retrieved from 164,591 Illumina effective sequences obtained from the 18 activated sludge samples, while the reads for archaeal phyla only accounted 1.22% of the total phyla.

**Fig. 1:**
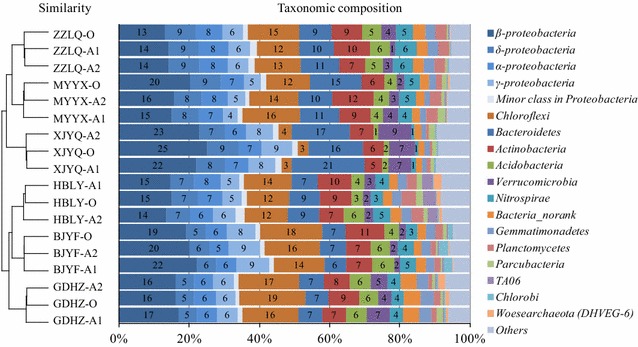
Percentages of the major phyla and major class in *Proteobacteria* in each WWTP (the sequence percentage is above 2% in at least one WWTP). Clustering on the left presented phylogenetic-based distances between the cases by unweighted UniFrac analysis. Unit of numbers: percentage (%)

The major phyla in each sample (the sequence percentage is above 2% in at least one WWTP) were *Proteobacteria*, *Chloroflexi*, *Bacteroidetes*, *Actinobacteria*, *Acidobacteria*, *Verrucomicrobia*, *Nitrospirae*, *Gemmatimonadetes*, *Planctomycetes*, *Parcubacteria*, *TA06*, *Chlorobi*, *Woesearchaeota* (*DHVEG*-*6*) and *Bacteria* (*norank*) (Fig. 1). *Proteobacteria* was the most dominant phylum and accounted 33.9–50.9% of all the phyla, followed by *Chloroflexi* (12.1–18.8%), *Bacteroidetes* (6.6–20.8%), *Actinobacteria* (5.1–11.8%), *Verrucomicrobia* (1.4–9.4%), *Acidobacteria* (1.4–6.3%) and *Nitrospirae* (0.8–6.1%). At the class level for *Proteobacteria*, *β*-*proteobacteria* represented 13.1–25.1% of total sequences, followed by *δ*-*proteobacteria* (5.2–9.0%), *α*-*Proteobacteria* (5.4–8.4%) and *γ*-*proteobacteria* (4.1–9.4%).

**Fig. 2:**
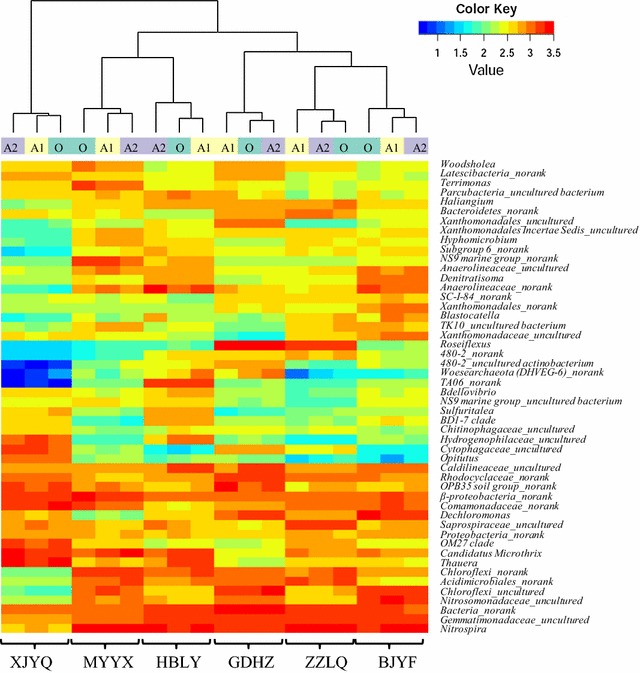
Heat map to profile the most abundant genera (top50) of the anaerobic-(A1), anoxic-(A2) and oxic-(O) zones in each WWTP. The *color bar* indicates the range of the percentage of a genus in a sample, based on the *color key* (log scale) at the *top right*

At the genus level, a total of 1594 genera were identified in the 18 activated sludge samples. Relative abundance of the top 50 genera from the anaerobic-(A1), anoxic-(A2) and oxic-(O) zones in the six WWTPs is shown in Fig. 2. These top 50 genera belong to 11 bacterial phyla (including *Nitrospirae*, *Proteobacteria* (*α*-, *β*-, *γ*-, *δ*- and norank), *Bacteroidetes*, *Gemmatimonadetes*, *Actinobacteria*, *Chloroflexi*, *Verrucomicrobia*, *Parcubacteria*, *Latescibacteria*, *TA06* and *Acidobacteria*) and an archaeal phylum of *Woesearchaeota* (*DHVEG*-*6*).

The most abundant genus is *Nitrospira* with a relative abundance varying from 0.8 to 6.1% in individual samples, while the second is *Gemmatimonadaceae* (belonging to *Gemmatimonadetes* phylum) with a relative abundance varying from 1.1 to 3.6% in individual samples. The other top 10 abundant genera detected in all samples were *Comamonadaceae* (*no rank*), *Thauera*, uncultured *Nitrosomonadaceae*, *Dechloromonas* and *Rhodocyclaceae* (*no rank*) belonging to *β*-*proteobacteria*, *Candidatus Microthrix* belonging to *Actinobacteria* pyhlum, *OPB35* soil group (*no rank*) belonging to *Verrucomicrobia* pyhlum, and *Chloroflexi* (*no rank and uncultured*) belonging to *Chloroflexi* phylum.

### Distribution of ammonium oxidizer organisms and denitrifiers

**Fig. 3:**
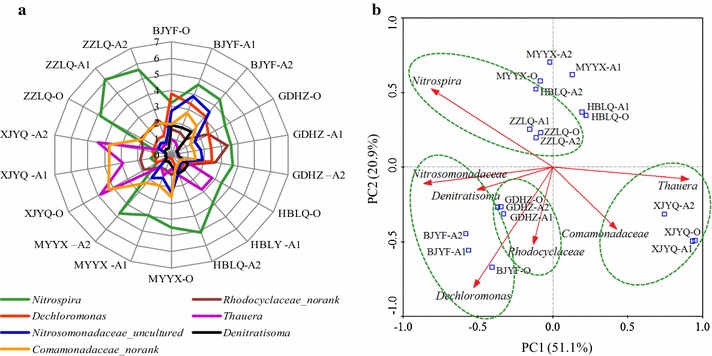
Distribution of ammonium oxidizers and denitrifiers of the 18 activated sludge samples. **a** Percentages of ammonium oxidizers and denitrifiers, **b** PCA based on the abundance

Nitrification and denitrification occur in Carrousel oxidation ditch wastewater treatment system for the catalysis of physiologically distinct clades of involved ammonium oxidizing bacteria (AOB) (oxidation of ammonium to nitrite), nitrite-oxidizing bacteria (NOB) (oxidation of nitrite to nitrate), and/or complete ammonium oxidizer (Comammox) (complete oxidation of ammonium to nitrate), and denitrifiers (reduction of nitrate via nitrite and intermediate gaseous nitrogen oxide products to dinitrogen). Illumina sequences associated with ammonium oxidizer organisms and denitrifiers of anaerobic-(A1), anoxic-(A2) and oxic-(O) activated sludge samples of the six WWTPs were shown in Fig. 3. *Nitrospira* (including detected *Candidatus Nitrospira defluvii* spp. and other two uncultured *Nitrospira* organisms) can conduct oxidation of nitrite to nitrate, with relative abundance over 3% of the activated sludge samples of the investigated WWTPs except for XJYQ. *Nitrosomonadaceae* (*uncultured*) as a group of the main contributors for oxidation of ammonium to nitrite occupied 1.5 to 3.8% of each sample from the WWTPs of BJYF, GDHZ and MYYX. The ratio of NOB to AOB [i.e. *Nitrospira*/*Nitrosomonadaceae* (*uncultured*)] ranged from 119 to 687% of all samples, and HBLY and ZZLQ might highlight the excellent nitrification process for the high ratios (406–687%).

Five denitrifying groups, *Dechloromonas*, *Thauera*, *Denitratisoma*, *Rhodocyclaceae* (norank) and *Comamonadaceae* (norank) with relative abundance ranged from 0.2 to 4.8% were detected in all the samples. *Dechloromonas*, *Thauera*, *Denitratisoma and Rhodocyclaceae* (norank) belongs to *Rhodocyclaceae* family.

PAC revealed that samples of anaerobic-(A1), anoxic-(A2) and oxic-(O) zone from each WWTP were grouping together, but the relative abundance of the ammonium oxidizers and denitrifers differed apparently with different WWTPs (Fig. 3b). Among the six WWTPs, the most relative abundant genera involved in nitrogen transformation in BJYF were *Dechloromonas* (3.1–3.8%), *Nitrosomonadaceae* (uncultured) (2.7–3.8%) and *Denitratisoma* (1.8%), and in XJYQ were *Thauera* (2.9–4.8%) and *Comamonadaceae* (norank) (3.7–4.3%), while that in ZZLQ and GDHZ exhibited *Nitrospira* (4.8–6.1%) and *Rhodocyclaceae* (norank) (2.5–3.4%), respectively. XJYQ presented the highest relative abundance of the sum of the five denitrifers among the six WWTPs, while MYYX was the lowest. The differences in wastewater quality and environmental factors may result in the bacterial abundant discrepancies among the WWTPs.

### Abundance differences between the 3 biological functional zones

**Fig. 4:**
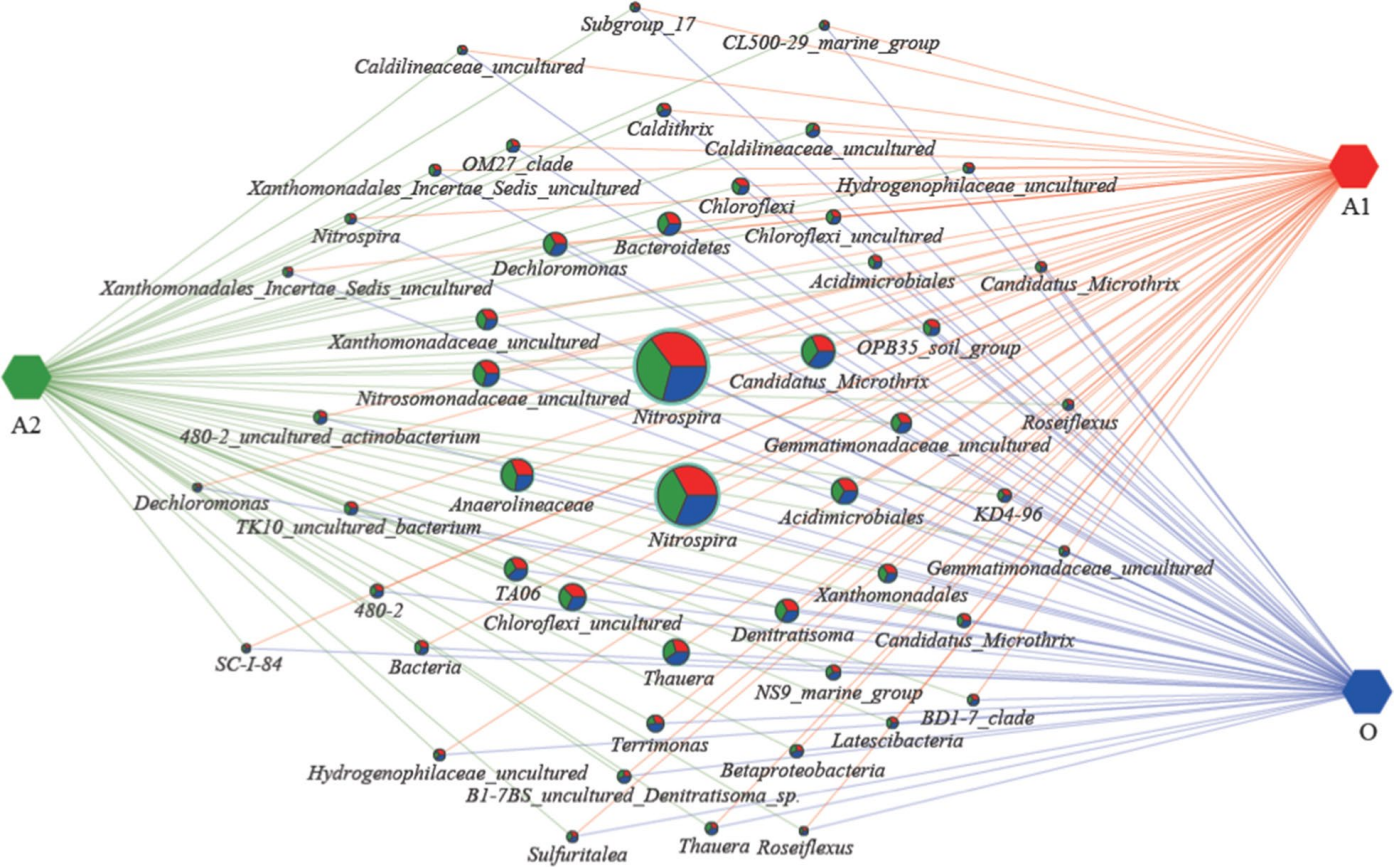
Profile clustering Cytoscape network visualize the top 50 abundant OTUs across the anaerobic-zone (A1) (*red*), anoxic-zone (A2) (*green*) and oxic-zone (O) (*blue*) in carrousel oxidization ditch system. Node sizes pie chart presented the relative abundances of the OTU over the respective samples

Clustering network analysis by Cytoscape was applied to gain a better insight into the differences of the anaerobic-, anoxic- and oxic-zones in carrousel oxidization ditch system. Figure 4 showed the most abundant 50 OTUs and presented the relative distribution and abundances between the 3 biological functional zones. *Nitrospira* (including *Candidatus Nitrospira defluvii* spp. and other two *uncultured Nitrospira* organisms) was the most abundant and ubiquitous bacterial genus with dominant occurrence among the 3 zones. The organisms involved in ammonium-oxidizing and denitrification as *Nitrosomonadaceae*, *Denitratisoma*, *Thauera*, *Dechloromonas* were also shared by the anaerobic-, anoxic- and oxic-zones with nearly the same percentage of the weighted-degree of the nod. Furthermore, the anaerobic-, anoxic- and oxic-zones shared approximately similar percentages across the 50 most abundant OTUs.

**Fig. 5:**
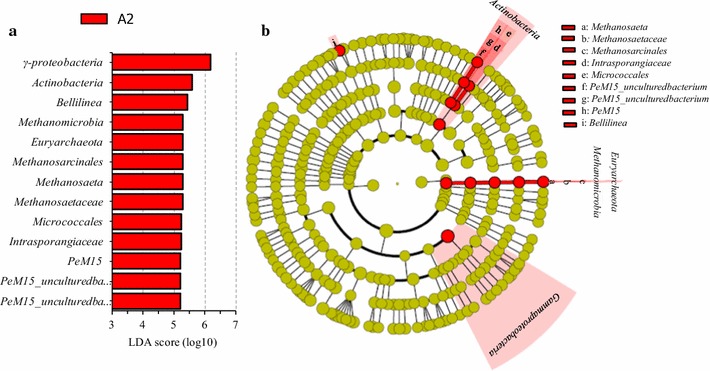
LEfSe analysis results of the anaerobic-, anoxic- and oxic-zones in carrousel oxidization ditch system. **a** LDA scores to feature differentially abundant, **b** Taxonomic representation of statistically and biologically consistent differences. Differences were indicated in the *color* of the most abundant taxa (*yellow* represented non-significant, and *red* indicated the anoxic-zone). *Each circle’s* diameter was proportional to the taxonomic abundance

The differentially abundant features among the anaerobic-, anoxic- and oxic-zones in carrousel oxidization ditch system were identified by LEfSe analysis (Fig. 5). The results showed that the taxa with significantly differential abundances were only detected from the anoxic-zone (A2). Figure 5a presented that 13 microbial clades showed statistically significant and biologically consistent differences in the anoxic-zone. The significantly differential abundant taxa in the anoxic-zone belonged to an archaeal phylum of *Euryarchaeota*, two bacterial classes of *Actinobacteria* and *γ*-*Proteobacteria* and an archaeal class of *Methanomicrobia*, and 3 genera of uncultured bacterium *PeM15*, *Methanosaeta* and *Bellilinea* (Fig. 5b). *γ*-*proteobacteria* had the highest LDA score, followed by *Actinobacteria* class and *Bellilinea* genus (within the phylum of *Chloroflexi*, class of *Anaerolineae*, order of *Anaerolineales* and family of *Anaerolineaceae*).

### Abundance differences between the geographically distributed Carrousel oxidization ditches

**Fig. 6:**
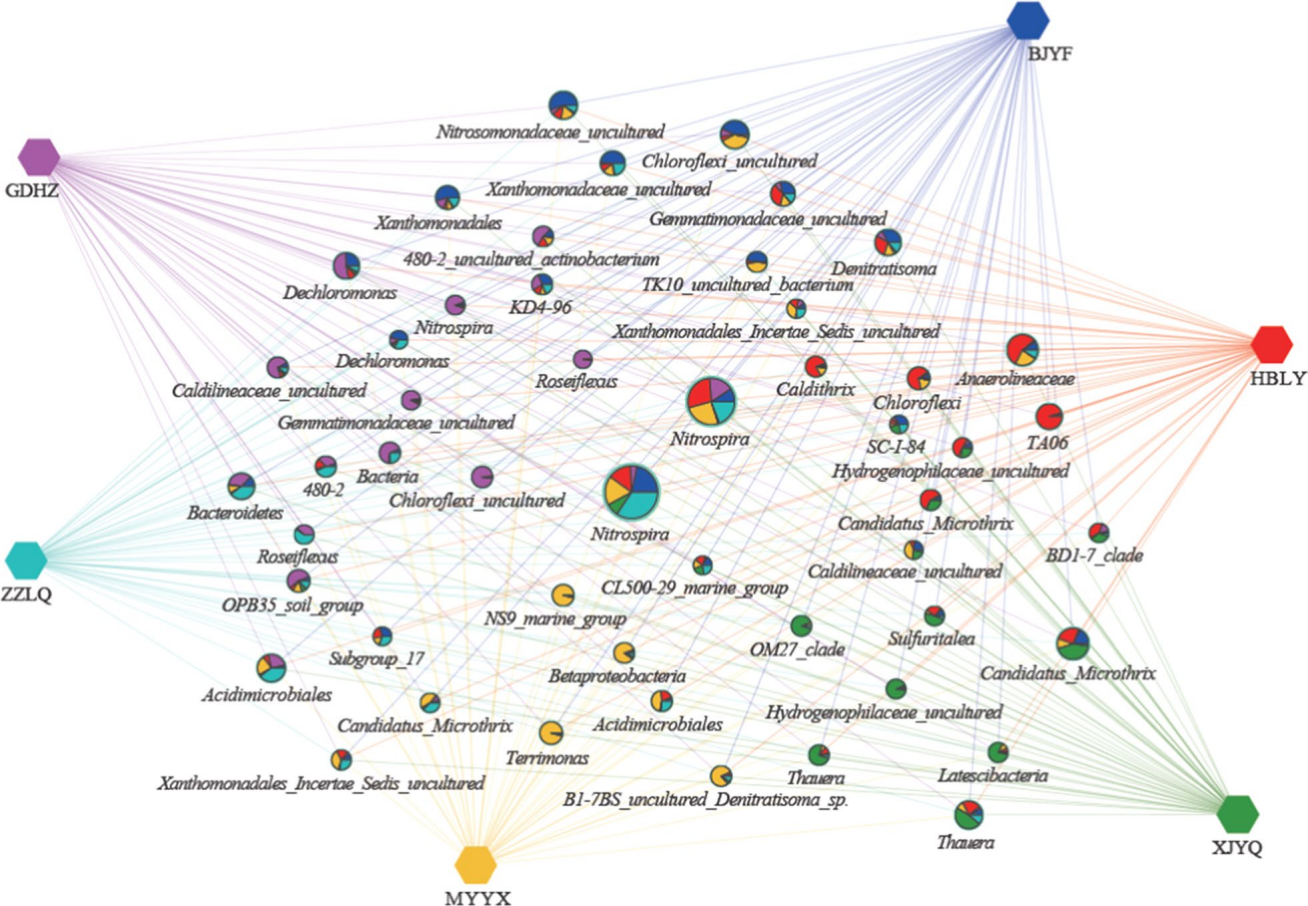
Profile clustering Cytoscape network visualize the top 50 abundant OTUs across the six WWTPs represented as XJYQ (*green*), ZZLQ (*medium aquamarine*), HBLY (*red*), BJYF (*blue*), GDHZ (*purple*) and MYYX (*yellow*), respectively. Node sizes pie chart presented the relative abundances of the OTU over the respective samples

Six full-scale Carrousel oxidization ditch WWTPs represented by XJYQ, ZZLQ, HBLY, BJYF, GDHZ and MYYX, which geographically locate at Northwest, Central South region, Central region, Southern and Southwest respectively, of China were investigated. The most abundant 50 OTUs and the relative distribution and abundances between the 6 WWTPs were analyzed by Cytoscape network analysis (Fig. 6). The dominant and ubiquitous bacterial genera among all the 6 WWTPs were *Nitrospira* (including *Candidatus Nitrospira defluvii* spp. and uncultured organism *Nitrospira*), *Candidatus Microthrix*, *Anaerolineaceae*, *uncultured Chloroflexi*, *uncultured Nitrosomonadaceae*, *Acidimicrobiales*, *Thauera*, *Dechloromonas* and *Denitratisoma*; however, the abundance proportion of each WWTP presented obvious discrepancy.

**Fig. 7:**
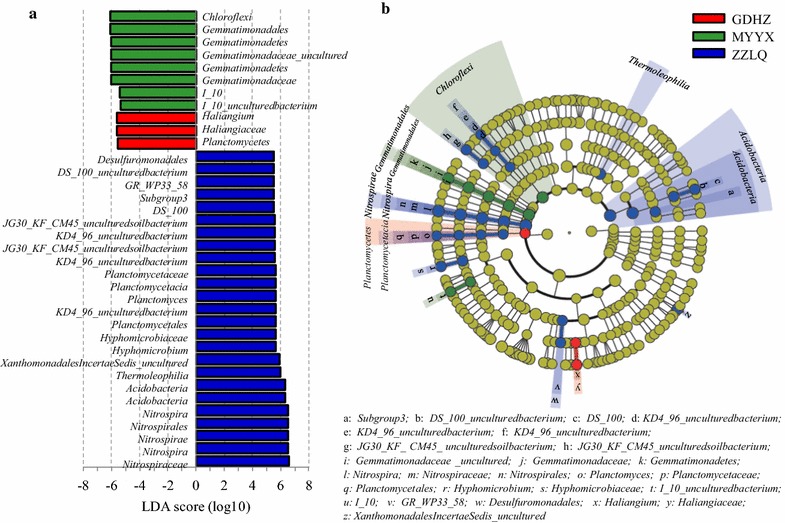
LEfSe analysis results of the WWTPs. **a** LDA scores to feature differentially abundant, **b** Taxonomic representation of statistically and biologically consistent differences. Differences were indicated in the *color* of the most abundant taxa (*yellow* represented non-significant, and *red*, *green* and *blue* indicated the WWTP of GDHZ, MYYX and ZZLQ, respectively). *Each circle’s* diameter was proportional to the taxonomic abundance

To gain insight into the differences of the six Carrousel oxidization ditches, LEfSe analysis was employed (Fig. 7). The results showed that the significant differential abundances occurred in three WWTPs i.e. GDZH, MYYX and ZZLQ, of which 36 differentially abundant taxonomic clades were found. Taxa of *Nitrospiraceae*, *Nitrospira*, *Nitrospirae*, *Nitrospirales and Acidobacteria* in ZZLQ had the highest LDA score among all OTUs. *Chloroflexi* and *Haliangium* presented the highest LDA score in the OTUs of MYYX and GDZH, respectively.

The most differentially abundant taxa in ZZLQ belonged to two bacterial phyla of *Nitrospirae* and *Actinobacteria*, while that in MYYX belonged to two bacterial phyla of *Chloroflexi* and *Gemmatimonadales*, and that in MYYX belonged to a bacterial phylum of *Planctomycetes* (Fig. 7b). At family level, 7 differentially abundant taxonomic clades were detected in ZZLQ (including *Hyphomicrobiaceae*, *Planctomycetaceae*, *Nitrospiraceae*, uncultured soil bacterium *JG30*-*KF*-*CM45*, uncultured bacterium *KD4*-*96*, *DS*-*100* and *GR*-*WP33*-*58*), while that of 2 in MYYX (*Gemmatimonadaceae* and *I*-*10*) and one in GDZH (*Haliangiaceae*). At genus level, also 7 differentially abundant taxonomic clades were detected in ZZLQ (including *Hyphomicrobium*, *Planctomyces*, *Nitrospira*, uncultured soil bacterium *JG30*-*KF*-*CM45*, uncultured bacterium *KD4*-*96*, *DS*-*100* and uncultured *XanthomonadalesIncertaeSedis*), while 2 differentially abundant taxonomic clades were detected in MYYX (*uncultured Gemmatimonadaceae* and uncultured bacterium *I*-*10*) and one in GDZH (*Haliangium*). Except the genera within the above phyla, 4 differentially abundant genera of *Hyphomicrobium* (within *Hyphomicrobiaceae* family and *Rhizobiales* order and *α*-*proteobacteria*), uncultured bacterium *I*-*10* (within *I*-*10* family and *Rhodospirillales* order and *α*-*proteobacteria*), *Haliangium* (within *Haliangiaceae* family and *δ*-*Proteobacteria* calss) and uncultured *XanthomonadalesIncertaeSedis* (within *Xanthomonadales* order, *γ*-*proteobacteria*) were also detected.

### The relationships between environmental variables and microbial community

**Fig. 8:**
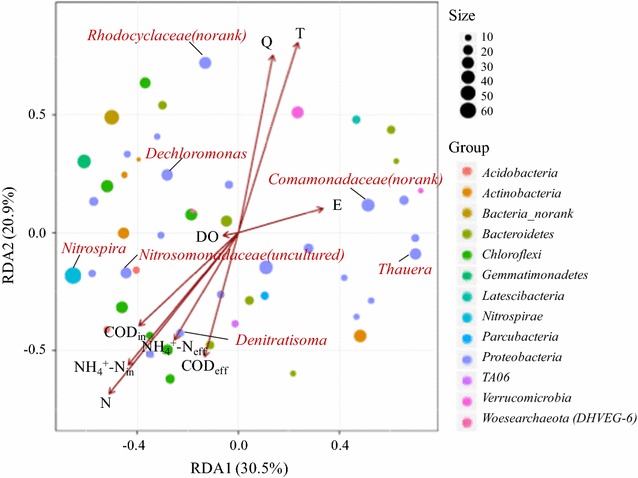
Redundancy analysis (RDA) of correlation between environmental variables and the most abundant genera from 18 samples. *Each circle* represents a genus and the size relates to its total relative abundance of all samples. Various *circle colors* indicate different phyla. The environmental variables including T, E, N, Q, DO, COD_in_, COD_eff_, NH_4_^+^-N_in_ and NH_4_^+^-N_eff_ represent water temperature, eastern longitude, northern latitude, water volume, dissolved oxygen, influent of COD, effluent of COD, influent of ammonium and effluent of ammonium of the WWTP, respectively

The relationship between environmental variables and the abundance of microbial community referred to the major genera (top50) relative abundances were identified by RDA (Fig. 8). Nine environmental variables including geographical location parameters (i.e. eastern longitude and northern latitude of the WWTP), operating parameters of each WWTP (i.e. water volume, influent/effluent concentration of COD and ammonium nitrogen), and physicochemical parameters of each sample (i.e. water temperature and dissolved oxygen) (Additional file 1: Table S1) were investigated. Arrows indicate the direction and magnitude of environmental variables associated with bacterial community structures. The results showed that the eastern longitude and dissolved oxygen were not as strongly correlated to microbial community composition as the other environmental variables. Six environmental variables, i.e. water temperature, northern latitude, water volume, influent ammonium nitrogen, influent COD and effluent COD exhibited significant correlation to microbial community according to the Monte Carlo permutation test analysis (*p* < 0.05). Also, water temperature and northern latitude were the crucial factors shaped the variations in microbial community composition. Among the genera of ammonium oxidizer organisms and denitrifiers, the abundance of *Nitrospira*, *Nitrosomonadaceae* (*uncultured*) and *Denitratisoma* presented strong positive correlations with the northern latitude and influent/effluent concentration of COD and ammonium nitrogen, respectively, while *Dechloromonas*, *Thauera*, *Rhodocyclaceae* (*norank*) and *Comamonadaceae* (*norank*) presented positive correlations with the water volume and temperature.

## Discussion

*Proteobacteria*, *Chloroflexi*, *Bacteroidetes*, *Actinobacteria*, *Verrucomicrobia*, *Acidobacteria* and *Nitrospirae* were dominating phyla across the anaerobic-, anoxic- and oxic-zones in Carrousel oxidation ditch system, and accounted 75.2–85.8% of all sequences of the six investigated full-scale WWTPs. *Proteobacteria* (mainly including *α*-, *β*-, *γ*-, *δ*-*proteobacteria*) was the most dominant phylum and accounted 33.9–50.9% of all the monitored phyla. Previous reports on activated sludge from municipal WWTPs have showed that the above phyla are common and *Proteobacteria*, *Actinobacteria*, *Bacteroidetes* and *Verrucomicrobia* dominated in both DNA and cDNA sets (Yu and Zhang 2012; Cydzik-Kwiatkowska and Zielińska 2016); also *Proteobacteria* commonly dominated 21–65% of all phyla and the *β*-*proteobacteria* is the most abundant class which is mainly responsible for organic and nutrient removal (Nielsen et al. 2010; Hu et al. 2012; Wang et al. 2014b; Cydzik-Kwiatkowska and Zielińska 2016). Compared to common wastewater treatment activated sludge system, the phylum *Nitrospirae*, of which members are usually found as predominant NOB in WWTPs (Nielsen et al. 2010; Rodríguez et al. 2015), presented in the subdominant phyla in the Carrousel oxidation ditch system for nitrogen removal function.

The genus *Nitrospira* was the most abundant and ubiquitous bacterial genus across the anaerobic-, anoxic- and oxic-zones in the Carrousel oxidation ditch systems. The second most abundant genus is *Gemmatimonadaceae*, which family members had also been detected in activated sludge sewage treatment systems, and could grow by both aerobic and anaerobic respiration (Zhang et al. 2003; Shu et al. 2015). The other subdominant genera were *Comamonadaceae* (*no rank*), *Thauera*, uncultured *Nitrosomonadaceae*, *Dechloromonas*, *Rhodocyclaceae* (no rank) *Candidatus Microthrix*, *OPB35* soil group (no rank) and *Chloroflexi* (no rank and uncultured). *Candidatus Microthrix* (ranging from 0.5 to 4.1% in individual samples) commonly occurred at the water–air interface of biological WWTPs, where they are considered notorious in causing foaming and bulking (Muller et al. 2012). Also the *Candidatus Microthrix parvicella* is been considered as a specialized lipid consumer with a physiological potential analogous to polyphosphate-accumulating organisms (PAOs) and glycogen accumulating organisms (GAOs) (Nielsen et al. 2002). The *Verrucomicrobia OPB35* soil group, the most abundant prokaryotic taxa (Lanzén et al. 2015), were also detected in the 18 activated samples with the abundance ranged from 0.5 to 3.4%. The relative abundance of *Chloroflexi* (no rank and uncultured) ranged from 0.2 to 4.9% of the samples. Members of phylum *Chloroflexi* are commonly detected from sediment and involve carbon cycling, organohalide respiration, fermentation, CO_2_ fixation, and acetogenesis with ATP formation by substrate-level phosphorylation (Hug et al. 2013).

Illumina sequences associated with ammonium oxidizer organisms and denitrifiers included *Nitrospira* (including *Candidatus Nitrospira defluvii* spp. and other two *uncultured Nitrospira* organisms), uncultured *Nitrosomonadaceae*, *Dechloromonas*, *Thauera*, *Denitratisoma*, *Rhodocyclaceae* (norank) and *Comamonadaceae* (norank), which presented top50 abundant genera in all samples. *Nitrospira* as the most diverse known group of NOB in classical two-step nitrification theory (oxidation of ammonium via nitrite to nitrate), in which of *Candidatus Nitrospira inopinata* is recently discovered to be complete ammonium oxidizer (Comammox) organisms i.e., novel one step nitrification theory which completely oxidize ammonium to nitrate (Daims et al. 2015; van Kessel et al. 2015). *Candidatus Nitrospira defluvii* spp., a predominant nitrite oxidizer in WWTP, possesses a periplasmically oriented enzyme nitrite oxidoreductase (NXR), which differs from other known nitrite oxidizers as *Nitrobacter* and *Nitrococcus* (Lucker et al. 2010; Nielsen et al. 2010; Rodríguez et al. 2015). The periplasmic forms of NXR are considered to be more efficient because of more proton-motive force produced by per oxidized nitrite and no nitrite/nitrate transporting across the cytoplasmic membrane (Lucker et al. 2010; Rodríguez et al. 2015). *Nitrosomonadaceae* (uncultured) presented a relative abundance varying from 0.27 to 3.8% in individual samples. Also, genus *Nitrosomonas* was detected in all the samples with relative low abundance of 0.03–0.35% (out of the top 50 genera). The genera within family *Nitrosomonadaceae* as *Nitrosomonas* and *Nitrosospira* are the most detected AOB in WWTPs (Li et al. 2016). The other dominant genera of *Comamonadaceae* (*no rank*), *Thauera*, *Dechloromonas* and *Rhodocyclaceae* (*no rank*) are capable of denitrification (Hwang et al. 2006; Lu et al. 2014). *Rhodocyclaceae* and *Comamonadaceae* (both within *β*-*proteobacteria*) were reported as the core families with responsibility for denitrifying and aromatic degrading processes in wastewater treatment activated sludge systems (Loy et al. 2005; Ma et al. 2015), of which *Thauera* is one of the most often detected genera and functionally important denitrifier in activated sludge systems (Lu et al. 2014; Ma et al. 2015). Furthermore, *Dechloromonas*-related bacteria have been demonstrated with the capability of nitrate/nitrite reduction, acetate uptake and polyphosphate and polyhydroxyacids storage (Cydzik-Kwiatkowska and Zielińska 2016). Among the anaerobic-(A1), anoxic-(A2) and oxic-(O) function zones, the abundances of AOB, NOB and denitrifiers clustered together within each Carrousel oxidation ditch system (Fig. 3b), because Carrousel oxidation ditch is a looped channel system, and the most of the activated sludge separated from the final clarifier is returned to the anaerobic-zone to maintain the proper biomass level in the system.

Furthermore, the phylogeny-based UniFrac analysis (Fig. 1), clustering network analysis (Fig. 4), and LEfSe analysis (Fig. 5) demonstrated that the activated sludge samples were certainly similar between those taken from the same type of plant (i.e. the different biological zones). The clustering network analysis showed that anaerobic-, anoxic- and oxic-zones shared approximately similar percentages across the 50 most abundant OTUs. The standard deviation (SD, calculated by weighted-degree of the three zones at a certain OTU) of the top 50 OTUs ranged from 0.2 to 14.3, and Coefficient of variation (CV, calculated as the ratio of the SD to the mean and multiplied by 100), evaluating dispersion of a probability distribution among the three zones, ranged from 3.0 to 19. Only 6 OTUs (referred to the genera of *Terrimonas*, *Caldilineaceae* (uncultured), *β*-*proteobacteria* (no rank), uncultured *B1*-*7BS*, *Denitratisoma*, *Caldithrix* and uncultured *Hydrogenophilaceae*) presented the CV value higher than 15, which indicated that the probability distributions of the 6 OTUs exhibited relative higher dispersion degree among the 3 biological functional zones. The LEfSe analysis result indicated only13 microbial clades within an archaeal phylum of *Euryarchaeota* and two bacterial classes of *γ*-*proteobacteria* (with the highest LDA score) and *Actinobacteria*, showed statistically significant differences in the anoxic-zone among the 3 biological zones (Fig. 5). At genus level, there were 3 differential abundant taxa (i.e. uncultured bacterium *PeM15*, *Methanosaeta* and *Bellilinea*) in the anoxic-zone among the 3 biological zones. The members of *Actinobacteria* phylum have been verified as important PAOs in enhanced biological phosphorus removal systems, and also some may contain nitrite reductase genes involved in denitrification (Rodríguez et al. 2015). *Methanosaeta* within *Methanosarcinales* is an obligate aceticlastic methanogen (Rodríguez et al. 2015). *Methanosarcinales* showed statistically significant differences in the anoxic-zone among the 3 biological zones might be caused by the high acetate concentrations (Anderson et al. 2009), which were added into the anoxic-zone as external carbon source to enhance the denitrification process.

The 6 studied biological WWTPs with Carrousel oxidization ditches process shared the major phyla (Fig. 1) and the most abundant genera (top50) (Fig. 2), but were dissimilar in the microbial abundance distribution. These results were complying with previous reported microbial community structure distribution in geographically distributed biological WWTPs (Xia et al. 2010; Shu et al. 2015). The distribution of detected AOB, NOB and denitrifiers also exhibited apparently differences among the 6 WWTPs (Fig. 3). The significant differential abundances occurred in three WWTPs i.e. GDZH, MYYX and ZZLQ by the LEfSe analysis (Fig. 7), which indicated that 36 microbial clades within 7 bacterial phyla of *Nitrospira*, *Planctomycetes*, *Gemmatimonadales*, *Chloroflexi*, *Acidobacteria* and *Proteobacteria* presented statistically significant differences. At genus level, there were 10 significantly differential abundant taxa, of which genera *Nitrospira*, *uncultured Gemmatimonadaceae* and *Haliangium* showed the highest LDA score in the activated sludge samples from the ZZLQ, MYYX and GDHZ, respectively. Such discrepancies in microbial community structures among the geographically distributed WWTPs harbored the same biological treatment process might be correlated with the different environmental conditions and operating parameters among the different geographically located WWTPs (Hu et al. 2012; Xia et al. 2016).

The RDA analytical results (Fig. 8) showed that the abundance of *Nitrospira*, uncultured *Nitrosomonadaceae* and *Denitratisoma* presented strong positive correlations with the northern latitude of the WWTP, and influent/effluent concentration of COD and ammonium nitrogen. *Dechloromonas*, *Thauera*, *Rhodocyclaceae* (*norank*) and *Comamonadaceae* (*norank*) presented positive correlations with the water volume and temperature. Among the tested environmental variables, the temperature and water quality play important parts in the microbial metabolisms, nitrifying process and denitrification (Ju et al. 2014; Lu et al. 2014; Wang et al. 2014a). The temperature and influent ammonium nitrogen can impact the abundance of AOB and the balance between AOB and NOB in activated sludge systems (Cydzik-Kwiatkowska and Zielińska 2016). Also, *Nitrospira* and *Nitrosomonas* have been verified more susceptible to seasonal variations and displayed higher abundances in relative higher temperature (Wan et al. 2011; Ju et al. 2014). In the present study, the abundance of *Nitrospira*, *Nitrosomonadaceae* (uncultured) and *Denitratisoma* were greatly influenced by not only the water quality, but also the northern latitude of the WWTP. Shanks et al. (2013) reported that city latitude could result in the variation of the sewage infrastructure community composition among cities with a wide range of geographic locations. Zhang et al. (2012) found that although some detected microbial genera existed in all WWTPs, the microbial composition of biomass differed with different geographically location. Thus, city latitude might impact the microbial community structures of untreated wastewaters, which further influence the microbial community of biological treated system of WWTP. Also, the effects of city latitude on microbial composition might due to the fact that the temperatures, technological system and water quality of wastewater treatment vary in different locations (Cydzik-Kwiatkowska and Zielińska 2016). The northern latitude of the WWTP presented significant correlation with the water quality (i.e. influent/effluent concentration of COD and ammonium nitrogen) in this study (Fig. 8). However, further study on spatial and temporal dynamics of ammonium oxidizer organisms and denitrifiers in activated sludge system is needed. In general, the established relationship between microbial community and environmental variables in different biologically functional zones of the six representative WWTPs at different geographical locations made the present work of potential use for evaluation of practical wastewater treatment processes.

## Additional file

**Additional file 1: Table S1.** Characteristics of the six representative full-scale WWTPs. **Table S2:** Raw and trimmed reads, Good’s coverage, Chao1, ACE, Shannon, Simpson, and plus numbers of OTUs of the activated sludge samples.

## Abbreviations

WWTPs: wastewater treatment plants
FISH: fluorescence in situ hybridization
COD: chemical oxygen demand
PCR: polymerase chain reaction
OTUs: operational taxonomic units
LDA: linear discriminant analysis
LEfSe: linear discriminant analysis effect size pipeline
PCA: principal component analysis
RDA: redundancy analysis
AOB: ammonium oxidizing bacteria
NOB: nitrite-oxidizing bacteria

## References

[CR1] Ammary B, Radaideh J (2005). Simultaneous nitrification and denitrification in an oxidation ditch plant. Chem Biochem Eng Q.

[CR2] Anderson IJ, Sieprawska-Lupa M, Lapidus A, Nolan M, Copeland A, Del Rio TG, Tice H, Dalin E, Barry K, Saunders E, Han C, Brettin T, Detter JC, Bruce D, Mikhailova N, Pitluck S, Hauser L, Land M, Lucas S, Richardson P, Whitman WB, Kyrpides NC (2009). Complete genome sequence of *Methanoculleus marisnigri* Romesser et al. 1981 type strain JR1. Stand Genomic Sci.

[CR3] Caporaso JG, Kuczynski J, Stombaugh J, Bittinger K, Bushman FD, Costello EK, Fierer N, Pena AG, Goodrich JK, Gordon JI (2010). QIIME allows analysis of high-throughput community sequencing data. Nat Methods.

[CR4] Clesceri LS, Greenberg AE, Eaton AD (1998). Standard methods for the examination of water and wastewater.

[CR5] Cydzik-Kwiatkowska A, Zielińska M (2016). Bacterial communities in full-scale wastewater treatment systems. World J Microbiol Biotechnol.

[CR6] Daims H, Lebedeva EV, Pjevac P, Han P, Herbold C, Albertsen M, Jehmlich N, Palatinszky M, Vierheilig J, Bulaev A, Kirkegaard RH, von Bergen M, Rattei T, Bendinger B, Nielsen PH, Wagner M (2015). Complete nitrification by *Nitrospira* bacteria. Nature.

[CR7] Edgar RC (2010). Search and clustering orders of magnitude faster than BLAST. Bioinformatics.

[CR8] Edgar RC, Haas BJ, Clemente JC, Quince C, Knight R (2011). UCHIME improves sensitivity and speed of chimera detection. Bioinformatics.

[CR9] Guo CZ, Fu W, Chen XM, Peng DC, Jin PK (2013). Nitrogen-removal performance and community structure of nitrifying bacteria under different aeration modes in an oxidation ditch. Water Res.

[CR10] Hu M, Wang X, Wen X, Xia Y (2012). Microbial community structures in different wastewater treatment plants as revealed by 454-pyrosequencing analysis. Bioresour Technol.

[CR11] Hug LA, Castelle CJ, Wrighton KC, Thomas BC, Sharon I, Frischkorn KR, Williams KH, Tringe SG, Banfield JF (2013). Community genomic analyses constrain the distribution of metabolic traits across the *Chloroflexi* phylum and indicate roles in sediment carbon cycling. Microbiome.

[CR12] Hwang C, Wu WM, Gentry TJ, Carley J, Carroll SL, Schadt C, Watson D, Jardine PM, Zhou J, Hickey RF, Criddle CS, Fields MW (2006). Changes in bacterial community structure correlate with initial operating conditions of a field-scale denitrifying fluidized bed reactor. Appl Microbiol Biotechnol.

[CR13] Jin L, Zhang G, Tian H (2014). Current state of sewage treatment in China. Water Res.

[CR14] Jin P, Wang X, Wang X, Ngo HH, Jin X (2015). A new step aeration approach towards the improvement of nitrogen removal in a full scale Carrousel oxidation ditch. Bioresour Technol.

[CR15] Ju F, Guo F, Ye L, Xia Y, Zhang T (2014). Metagenomic analysis on seasonal microbial variations of activated sludge from a full-scale wastewater treatment plant over 4 years. Environ Microbiol Rep.

[CR16] Lanzén A, Epelde L, Garbisu C, Anza M, Martín-Sánchez I, Blanco F, Mijangos I (2015). The community structures of prokaryotes and fungi in mountain pasture soils are highly correlated and primarily influenced by pH. Front Microbiol.

[CR17] Lei L, Ni J (2014). Three-dimensional three-phase model for simulation of hydrodynamics, oxygen mass transfer, carbon oxidation, nitrification and denitrification in an oxidation ditch. Water Res.

[CR18] Li X, Sun S, Badgley BD, Sung S, Zhang H, He Z (2016). Nitrogen removal by granular nitritation-anammox in an upflow membrane-aerated biofilm reactor. Water Res.

[CR19] Liu YC, Shi HC, Xia L, Shi HM, Shen TG, Wang ZQ, Wang G, Wang YZ (2010). Study of operational conditions of simultaneous nitrification and denitrification in a Carrousel oxidation ditch for domestic wastewater treatment. Bioresour Technol.

[CR20] Loy A, Schulz C, Lücker S, Schöpfer-Wendels A, Stoecker K, Baranyi C, Lehner A, Wagner M (2005). 16S rRNA gene-based oligonucleotide microarray for environmental monitoring of the *betaproteobacterial* order ‘‘*Rhodocyclales*’’. Appl Environ Microbiol.

[CR21] Lu H, Chandran K, Stensel D (2014). Microbial ecology of denitrification in biological wastewater treatment. Water Res.

[CR22] Lucker S, Wagner M, Maixner F, Pelletier E, Koch H, Vacherie B, Rattei T, Damste JS, Spieck E, Le Paslier D, Daims H (2010). A *Nitrospira* metagenome illuminates the physiology and evolution of globally important nitrite-oxidizing bacteria. Proc Natl Acad Sci USA.

[CR23] Ma Q, Qu Y, Shen W, Zhang Z, Wang J, Liu Z, Li D, Li H, Zhou J (2015). Bacterial community compositions of coking wastewater treatment plants in steel industry revealed by Illumina high-throughput sequencing. Bioresour Technol.

[CR24] Masella AP, Bartram AK, Truszkowski JM, Brown DG, Neufeld JD (2012). PANDAseq: paired-end assembler for illumina sequences. BMC Bioinform.

[CR25] Muller EE, Pinel N, Gillece JD, Schupp JM, Price LB, Engelthaler DM, Levantesi C, Tandoi V, Luong K, Baliga NS, Korlach J, Keim PS, Wilmes P (2012). Genome sequence of “*Candidatus Microthrix parvicella*” Bio17-1, a long-chain-fatty-acid-accumulating filamentous *actinobacterium* from a biological wastewater treatment plant. J Bacteriol.

[CR26] Nielsen PH, Roslev P, Dueholm TE, Nielsen JL (2002). *Microthrix parvicella*, a specialized lipid consumer in anaerobic–aerobic activated sludge plants. Water Sci Technol.

[CR27] Nielsen PH, Mielczarek AT, Kragelund C, Nielsen JL, Saunders AM, Kong Y, Hansen AA, Vollertsen J (2010). A conceptual ecosystem model of microbial communities in enhanced biological phosphorus removal plants. Water Res.

[CR28] Peng YZ, Hou HX, Wang SY (2008). Nitrogen and phosphorus removal in pilot scale anaerobic-anoxic oxidation ditch system. J Environ Sci.

[CR29] Rodríguez E, García-Encina PA, Stams AJM, Maphosa F, Sousa DZ (2015). Meta-omics approaches to understand and improve wastewater treatment systems. Rev Environ Sci Biotechnol.

[CR30] Saida BA, Latifa H, Hayet C, Hedi D (2010). Aeration management in an oxidation ditch. Desalination.

[CR31] Segata N, Izard J, Waldron L, Gevers D, Miropolsky L, Garrett WS, Huttenhower C (2011). Metagenomic biomarker discovery and explanation. Genome Biol.

[CR32] Shanks OC, Newton RJ, Kelty CA, Huse SM, Sogin ML, McLellan SL (2013). Comparison of the microbial community structures of untreated wastewaters from different geographic locales. Appl Environ Microbiol.

[CR33] Shannon P, Markiel A, Ozier O, Baliga NS, Wang JT, Ramage D, Amin N, Schwikowski B, Ideker T (2003). Cytoscape: a software environment for integrated models of biomolecular interaction networks. Genome Res.

[CR34] Shu D, He Y, Yue H, Wang Q (2015). Microbial structures and community functions of anaerobic sludge in six full-scale wastewater treatment plants as revealed by 454 high-throughput pyrosequencing. Bioresour Technol.

[CR35] van Kessel MA, Speth DR, Albertsen M, Nielsen PH, den Camp HJO, Kartal B, Jetten MS, Lücker S (2015). Complete nitrification by a single microorganism. Nature.

[CR36] Vanwonterghem I, Jensen PD, Ho DP, Batstone DJ, Tyson GW (2014). Linking microbial community structure, interactions and function in anaerobic digesters using new molecular techniques. Curr Opin Biotechnol.

[CR37] Wan CY, De Wever H, Diels L, Thoeye C, Liang JB, Huang LN (2011). Biodiversity and population dynamics of microorganisms in a full-scale membrane bioreactor for municipal wastewater treatment. Water Res.

[CR38] Wang Q, Garrity GM, Tiedje JM, Cole JR (2007). Naive Bayesian classifier for rapid assignment of rRNA sequences into the new bacterial taxonomy. Appl Environ Microbiol.

[CR39] Wang X, Xia Y, Wen X, Yang Y, Zhou J (2014). Microbial community functional structures in wastewater treatment plants as characterized by GeoChip. PLoS ONE.

[CR40] Wang Z, Zhang XX, Lu X, Liu B, Li Y, Long C, Li A (2014). Abundance and diversity of bacterial nitrifiers and denitrifiers and their functional genes in tannery wastewater treatment plants revealed by high-throughput sequencing. PLoS ONE.

[CR41] Xia S, Duan L, Song Y, Li J, Piceno YM, Andersen GL, Alvarez-Cohen L, Moreno-Andrade I, Huang CL, Hermanowicz SW (2010). Bacterial community structure in geographically distributed biological wastewater treatment reactors. Environ Sci Technol.

[CR42] Xia Y, Hu M, Wen X, Wang X, Yang Y, Zhou J (2016). Diversity and interactions of microbial functional genes under differing environmental conditions: insights from a membrane bioreactor and an oxidation ditch. Sci Rep.

[CR43] Xie W, Zhang R, Li W, Ni B, Fang F, Sheng G, Yu H, Song J, Le D, Bi X, Liu C, Yang M (2011). Simulation and optimization of a full-scale Carrousel oxidation ditch plant for municipal wastewater treatment. Biochem Eng J.

[CR44] Yang M, Sun P, Wang R, Han J, Wang J, Song Y, Cai J, Tang X (2013). Simulation and optimization of ammonium removal at low temperature for a double channel oxidation ditch based on fully coupled activated sludge model (FCASM): a full-scale study. Bioresour Technol.

[CR45] Yu K, Zhang T (2012). Metagenomic and metatranscriptomic analysis of microbial community structure and gene expression of activated sludge. PLoS ONE.

[CR46] Zhang H, Sekiguchi Y, Hanada S, Hugenholtz P, Kim H, Kamagata Y, Nakamura K (2003). *Gemmatimonas aurantiaca* gen. nov., sp. nov., a gram-negative, aerobic, polyphosphate-accumulating micro-organism, the first cultured representative of the new bacterial phylum *Gemmatimonadetes* phyl. nov. Int J Syst Evol Microbiol.

[CR47] Zhang T, Shao MF, Ye L (2012). 454 pyrosequencing reveals bacterial diversity of activated sludge from 14 sewage treatment plants. SME J.

[CR48] Zhang C, Li S, Yang L, Huang P, Li W, Wang S, Zhao G, Zhang M, Pang X, Yan Z, Liu Y, Zhao L (2013). Structural modulation of gut microbiota in life-long calorie-restricted mice. Nat Commun.

[CR49] Zhang QH, Yang WN, Ngo HH, Guo WS, Jin PK, Dzakpasu M, Yang SJ, Wang Q, Wang XC, Ao D (2016). Current status of urban wastewater treatment plants in China. Environ Int.

[CR50] Zheng M, Tian Y, Liu T, Ma T, Li L, Li C, Ahmad M, Chen Q, Ni J (2015). Minimization of nitrous oxide emission in a pilot-scale oxidation ditch: generation, spatial variation and microbial interpretation. Bioresour Technol.

[CR51] Zhou X, Han Y, Guo X (2015). Identification and evaluation of SND in a full-scale multi-channel oxidation ditch system under different aeration modes. Chem Eng J.

