# Supplementary material for: Microbial community compositions in different functional zones of Carrousel oxidation ditch system for domestic wastewater treatment
Source: AMB Express. 2017 Feb 15;7:40. doi: 10.1186/s13568-017-0336-y (PMC5311017; doi:10.1186/s13568-017-0336-y)
Supplement: Supplementary file 1 — Additional file 1: Table S1. Characteristics of the six representative full-scale WWTPs. Table S2: Raw and trimmed reads, Good’s coverage, Chao1, ACE, Shannon, Simpson, and plus numbers of OTUs of the activated sludge samples. [file 13568_2017_336_MOESM1_ESM.docx]

**Supplementary Material**

**Journal: *AMB Express***

**Microbial community compositions in different functional zones of Carrousel oxidation ditch system for domestic wastewater treatment**

Dong Xu, Sitong Liu, Qian Chen, Jinren Ni*

Department of Environmental Engineering, Peking University; The Key Laboratory of Water and Sediment Sciences, Ministry of Education, Beijing 100871, China

***Corresponding author:**

Jinren Ni

Address:

Department of Environmental Engineering, Peking University, Beijing 100871, China

Tel.: +86-10-62751185

Fax: +86-10-62756526

E-mail address: [nijinren@iee.pku.edu.cn](mailto:nijinren@iee.pku.edu.cn)

**Table S1** Characteristics of the six representative full-scale WWTPs .

| WWTPs | Geographical Location | | | COD (mg/L) | | NH_4_^+^-N (mg/L) | | Flow rate  (m^3^/h) | T  (^o^C) |
| --- | --- | --- | --- | --- | --- | --- | --- | --- | --- |
|  | City /  Province | Eastern longitude(E) | Northern latitude (N) | Influent | Effluent | Influent | Effluent |  |  |
| XJYQ | Yanqi /  Xijiang | 85°13′ | 41°45′ | 227 | 20 | 45 | 3.2 | 625 | 21 |
| ZZLQ | Zhuzhou /  Hunan | 113°10′ | 27°50′ | 180 | 22 | 26 | 0.8 | 1667 | 29.8 |
| HBLY | Hebi /  Henan | 113°59′ | 35°26′ | 340 | 32.4 | 33.5 | 1.3 | 1042 | 17.7 |
| BJYF | Haidian /  Beijing | 116°03′ | 39°53′ | 150 | 25 | 40 | 1.4 | 417 | 22 |
| GDHZ | Huizhou /  Gangdong | 114°24′ | 23°5′ | 103.41 | 17.86 | 10.88 | 0.56 | 1250 | 28 |
| MYYX | Mianyang /  Sichuan | 103°45′ | 30°42′ | 222 | 18.2 | 13.9 | 0.28 | 1042 | 23 |

**Table S2** Raw and trimmed reads, Good’s coverage, Chao1, ACE, Shannon, Simpson, and plus numbers of OTUs of the activated sludge samples.

| Sample ID | Raw reads | Trimed reads | Good’s coverage | Chao 1 | ACE | Shannon | Simpson | OTUs |
| --- | --- | --- | --- | --- | --- | --- | --- | --- |
| BJYF-A1 | 145593 | 127607 | 89.4% | 21187.4 | 23577.6 | 10.6 | 0.9967 | 9458 |
| BJYF-A2 | 211064 | 184387 | 89.6% | 20716.2 | 22825.1 | 10.6 | 0.9969 | 9333 |
| BJYF-O | 214064 | 187729 | 89.4% | 21047.7 | 23190.9 | 10.7 | 0.9975 | 9601 |
| HBLY-A1 | 212082 | 185895 | 90.6% | 19516.6 | 21806.1 | 10.1 | 0.9958 | 8255 |
| HBLY-A2 | 215435 | 188482 | 90.0% | 20201.5 | 22753.6 | 10.2 | 0.9953 | 8741 |
| HBLY-O | 220075 | 193365 | 90.7% | 18936.2 | 21315.2 | 10.1 | 0.9956 | 8292 |
| XJYQ-A1 | 257621 | 230608 | 90.5% | 17293.8 | 19789.8 | 10.4 | 0.9972 | 8390 |
| XJYQ-A2 | 261410 | 234329 | 91.1% | 17068.8 | 19188.2 | 10.3 | 0.9969 | 8017 |
| XJYQ-O | 234576 | 210839 | 90.5% | 17519.0 | 20058.2 | 10.4 | 0.9969 | 8430 |
| MYYX-A1 | 184037 | 165490 | 89.4% | 21086.1 | 23486.6 | 10.5 | 0.9966 | 9325 |
| MYYX-A2 | 271784 | 244067 | 89.0% | 22201.3 | 24386.8 | 10.5 | 0.9967 | 9559 |
| MYYX-O | 228941 | 205804 | 88.5% | 22889.1 | 24791.3 | 10.4 | 0.9955 | 9658 |
| ZZLQ-A1 | 240967 | 218463 | 86.8% | 23535.1 | 26654.7 | 11.0 | 0.9968 | 10681 |
| ZZLQ-A2 | 232060 | 209993 | 87.7% | 23213.2 | 25966.1 | 10.9 | 0.9970 | 10109 |
| ZZLQ-O | 241668 | 218574 | 88.2% | 23089.2 | 26141.1 | 10.8 | 0.9972 | 9868 |
| GDHZ-A1 | 208168 | 180779 | 90.1% | 19051.0 | 21318.3 | 10.5 | 0.9969 | 9016 |
| GDHZ-A2 | 213331 | 185989 | 90.6% | 18308.5 | 20590.0 | 10.4 | 0.9967 | 8737 |
| GDHZ-O | 203726 | 177473 | 89.8% | 19182.5 | 21641.9 | 10.5 | 0.9967 | 9121 |
